# The effects of 10,000 voluntary contractions over 8 weeks on the strength of very weak muscles in people with spinal cord injury: a randomised controlled trial

**DOI:** 10.1038/s41393-020-0439-1

**Published:** 2020-02-21

**Authors:** Lydia W. Chen, Joanne V. Glinsky, Md. Shofiqul Islam, Muzaffor Hossain, Claire L. Boswell-Ruys, Chitra Kataria, Jason Redhead, Yuan Xiong, Emilie Gollan, Punam D. Costa, Sophie Denis, Marsha Ben, Lovely Chaudhary, Jun Wang, Md. Abu Khayer Hasnat, Jayne Yeomans, Simon C. Gandevia, Lisa A. Harvey

**Affiliations:** 1 John Walsh Centre for Rehabilitation Research, Faculty of Medicine and Health Sciences, University of Sydney, St Leonards, NSW Australia; 2 Spinal Injuries Unit, Royal North Shore Hospital, St Leonards, NSW Australia; 3 Centre for the Rehabilitation of the Paralysed, Savar, Dhaka Bangladesh; 4 Spinal Injuries Unit, Prince of Wales Hospital, Randwick, NSW Australia; 5 Neuroscience Research Australia (NeuRA), University of New South Wales, Randwick, NSW Australia; 6 Indian Spinal Injuries Centre, Sector—C, Vasant Kunj, Delhi, India; 7 Spinal Injuries Unit, Royal Rehab, Ryde, NSW Australia; 8 Guangdong Work Injury Rehabilitation Hospital, Guangdong, China; 9 Queensland Spinal Cord Injuries Service, Metro South Health, Brisbane, QLD Australia; 10 NSW Spinal Outreach Service, Ryde, NSW Australia

**Keywords:** Spinal cord diseases, Rehabilitation

## Abstract

**Study design:**

A multi-centred, single-blinded randomised controlled trial.

**Objectives:**

To determine the effect of 10,000 voluntary contractions over 8 weeks on the strength of very weak muscles in people with spinal cord injury (SCI).

**Settings:**

Seven hospitals in Australia and Asia.

**Methods:**

One hundred and twenty people with recent SCI undergoing inpatient rehabilitation were randomised to either a Treatment or Control Group. One major muscle group from an upper or lower limb was selected if the muscle had grade 1 or grade 2 strength on a standard six-point manual muscle test. Participants allocated to the Treatment Group performed 10,000 isolated contractions of the selected muscle group, as well as usual care in 48 sessions over 8 weeks. Participants allocated to the Control Group received usual care alone. Participants were assessed at baseline and 8 weeks by a blinded assessor. The primary outcome was voluntary muscle strength on a 13-point manual muscle test. There were three secondary outcomes capturing therapists’ and participants’ perceptions of strength and function.

**Results:**

The mean between-group difference of voluntary strength at 8 weeks was 0.4/13 points (95% confidence interval −0.5 to 1.4) in favour of the Treatment Group. There were no notable between-group differences on any secondary outcome.

**Conclusion:**

Ten thousand isolated contractions of very weak muscles in people with SCI over 8 weeks has either no or a very small effect on voluntary strength.

## Introduction

An important goal of rehabilitation following spinal cord injury (SCI) is to improve the voluntary strength of muscles directly affected by the injury [1–3]. SCI results in complete or partial disruption of descending motor pathways resulting in paralysis or weakness, respectively [4]. In addition, the loss of central activation and disuse results in muscle atrophy and further weakness [4]. There are many different interventions aimed at increasing voluntary strength in weak muscles following SCI, but surprisingly little evidence about their effectiveness [5–10]. The only exception to this is progressive resistance training [5, 7, 11–13]. Progressive resistance training requires muscles to contract five to ten times against a maximal load and to the point of fatigue [14, 15]. This is typically done a couple of times per training session, with training repeated 2–3 times per week [16–18]. While progressive resistance training appears to be effective for stronger muscles (i.e., those muscles able to actively move through full range of motion (ROM) against gravity), our work suggests that it may not be effective for weaker muscles where strength is directly affected by SCI (i.e., muscles unable to actively move through full ROM against gravity) [11, 19]. In addition, it is often challenging to use the principles of progressive resistance training in very weak muscles because it is difficult to fine tune the resistance to ensure fatigue after five to ten contractions. For this reason among others, clinicians often train very weak muscles by asking patients to do large numbers of repeated contractions either isometrically or through the angular range [20]. This type of training is also based on theories about neural plasticity and the need for repetitious movement to prompt neural recovery [10, 20].

Despite the widespread use of repeated contractions to increase the strength of very weak muscles, there is little evidence to show that this is effective. Interestingly, interventions such as body-weight supported treadmill training and robotic gait training, also involve repeated contractions of very weak muscles, albeit during cyclic movement of the legs [20]. A recent review of this literature [5] did not find any evidence to indicate that any type of gait training programmes involving repeated contractions increased strength although there was some suggestion that robotic gait training improved strength more than overground gait training [21, 22]. These results may in part reflect a low number of studies and insufficient participants to detect a treatment effect. Nonetheless, we need a better understanding of whether repeated contractions per se increase strength particularly given the evidence in stroke and brain injury suggesting that this training stimulus is effective [23].

Therefore, the primary aim of this study was to determine the effect of 10,000 voluntary contractions over 8 weeks on the strength of very weak muscles in people with SCI. For this purpose, we compared repeated voluntary contractions and usual care with usual care alone. We chose 10,000 contractions because this equated to 200 contractions a day over 8 weeks, and this was as much as we thought could be reasonably expected of people with SCI (and the therapists administering the treatment). We also looked at the effect of this intervention on participants’ perceptions of change in strength, participants’ perceptions of change in function, and therapists’ predictions of final strength.

## Method

A multi-centre assessor blinded randomised controlled trial was conducted in seven SCI units. Four SCI units were in Australia and the remaining three were in Bangladesh, India and China. The first and last participants were randomised in February 2017 and December 2018, respectively. The start of recruitment at the seven SCI units was staggered with the last SCI unit commencing recruitment in April 2018. The trial was prospectively registered with the Australian and New Zealand Clinical Trials Registry (ACTRN12617000115336). All applicable institutional and governmental regulations concerning the ethical use of human volunteers were followed, and the study was approved through all appropriate ethics committees.

One hundred and twenty inpatients with recent SCI were recruited from a series of patients admitted to one of the included SCI units. Participants were included in the study if they had any type of SCI, had sustained their SCI within the preceding 6 months and had weakness (less than grade 3 strength but at least grade 1 strength on the standard six-point manual muscle test) in one of the following target muscle groups on one side of the body: elbow flexors or extensors, wrist flexors or extensors, knee flexors or extensors, ankle dorsiflexors or plantar flexors. Participants were required to be an inpatient of one of the participating SCI units for the duration of their involvement in the trial (i.e., ~9 weeks). If participants were likely to be discharged, they needed to live close to the hospital so they could continue to receive daily training as an outpatient. In addition, participants were included if they were 16 years or over and were free of any other neurological condition or injury. Participants were excluded if they had any condition that prevented testing or training of the target muscle, were unable to co-operate (e.g., a serious medical condition or cognitive impairment) or did not speak the local language sufficiently well to provide informed consent. If participants had more than one muscle group that fitted the inclusion criteria, the target muscle group was chosen prior to randomisation by the treating therapist in consultation with the participant.

A secure random-allocation schedule was computer generated prior to commencement of the trial by an independent person not involved in the recruitment of participants. Randomisation was stratified by site and stored in opaque, sequentially numbered and sealed envelopes kept at a central off-site location. Once a participant was deemed suitable for the trial and baseline assessments were completed, an envelope was opened off-site and allocation revealed. The participant was considered to have entered the trial at this point in time. Eligible participants were randomly allocated to either a Treatment Group or Control Group.

### Treatment Group

Participants allocated to the Treatment Group received usual care but also performed ~10,000 voluntary contractions of the target muscle group over an 8-week period. These were performed as 200 contractions per day, six times a week for 8 weeks under supervision (i.e., 48 training sessions with a total of 9600 contractions). The supervision was provided by a physiotherapist, physiotherapy assistant or physiotherapy student on 5 days of the week, and by either a physiotherapist, physiotherapy assistant or family member/carer on the 6th day. During all training sessions, participants were encouraged to maximally contract their muscles for 2 s with a 2-s rest between each contraction. Longer rests were provided at any stage if required, but participants were encouraged to complete all 200 contractions, and were provided with verbal encouragement throughout. Each session was completed within a 30-min time frame. Where appropriate, participants were provided with auditory (i.e., using a metronome) and visual cues (e.g., markers were placed at the extremes of range and participants were encouraged to touch the markers) to help participants with the timing of the contractions and to ensure they moved through their full available ROM. Mechanical counters were also used to monitor the number of repetitions.

The strength training was progressed. Initially, participants who were very weak and unable to move through a full ROM with gravity eliminated were encouraged to focus on increasing their ROM during the contractions. Once a participant could readily move 200 times through a full ROM with gravity eliminated, they were required to move against gravity. Once participants could move 200 times through full ROM against gravity then resistance was introduced and gradually progressed as deemed appropriate by the treating therapist, with the focus on completing the 200 contractions. Resistance was provided manually or using a variety of equipment such as free weights and resistance bands. The details of the training provided to participants were recorded by therapists in training diaries. Participants were also encouraged to practice contracting the target muscle group as much as possible in their own time. Participants told their therapists of any additional training that was performed. The therapists were responsible for recording this in diaries. Participants allocated to the Treatment Group continued to receive usual care (as outlined below).

### Control Group

Participants allocated to the Control Group received usual care but were not permitted to receive more than two sessions per week of progressive resistance training (two sets of ten contractions) for the target muscle group and only if deemed necessary by the treating therapist.

### Usual care provided to both groups

This involved comprehensive functional training for activities of daily living as considered necessary by participants and their treating therapists (e.g., training to transfer, walk, roll and push a wheelchair), as well as other forms of therapy appropriate for managing fitness, respiratory compromise, contractures, spasticity and pain. Participants also received any type of strength training programme deemed appropriate by their treating therapists to all muscles with the exception of the target muscle group. Electrical stimulation was permitted provided it was not administered to the target muscle group.

### Assessment

Participants were measured once prior to randomisation and then again 8 weeks after randomisation. The assessors were blinded and the success of blinding was recorded. The primary outcome was muscle strength of the target muscle group. The secondary outcomes were participants’ perceptions of strength and function, and therapists’ predictions of final strength. The details of the outcome measures are as follows:

#### Muscle strength

A 13-point manual muscle test was used to measure maximal voluntary muscle strength of the target muscle group. This scale was adapted from the traditional six-point manual muscle test with pluses and minuses, and has been recently tested for reliability. The weighted kappa coefficient (95% confidence interval (CI)) reflecting the agreement between the two strength assessments by two different assessors for the wrist extensors and elbow flexors were 0.96 (0.93 to 0.99) and 0.94 (0.89 to 0.99), respectively [24]. A between-group difference of 1 point on the 13-point scale was set as the minimally worthwhile treatment effect prior to the commencement of the trial.

#### Participants’ perceptions of strength

At the completion of the trial, participants were asked to rate their impression of change in strength of the target muscle group on a 15-point scale where −7 indicated “*a very great deal worse*”, 0 indicates “*no change*” and +7 indicated “*a very great deal better*” [25]. For all analyses, the results were transformed onto a 15-point scale where 0 reflected −7 (on the original scale), 7 reflected 0 and 15 reflected +7.

#### Participants’ perceptions of function

At the completion of the trial, participants were asked to rate their impression of change in their abilities to use the target muscle group for functional activities on a 15-point scale where −7 indicated “*a very great deal worse*”, 0 indicated “*no change*” and +7 indicated “*a very great deal better*” for the target muscle group [25]. The results were transformed onto a 15-point scale for analyses, as above.

#### Difference between predicted and measured final strength

Prior to the baseline assessment, the treating therapists were asked to predict the likely strength of the target muscle group (on a 13-point manual muscle test) at the end of the 8-week study period. Then, after 8 weeks, a score was derived that reflected the difference between predicted and measured final strength. This difference was used in the analyses and captured any increases or decreases in strength over and above what therapists predicted after taking into account the initial neurological status of the participant and any other factors that therapists deemed important (e.g., willingness to exercise, age, other comorbidities and complications).

Finally, participants from the Treatment Group were asked to rate the burden of the training on an eleven-point scale, where 0 indicated that the training was “*not at all burdensome*” and 10 indicated that the training was “*extremely burdensome*”. These data were intended to provide some indication of the burden of the treatment. The burden of treatment was not an outcome measure and these data were only analysed descriptively.

### Statistical analysis

The power calculation indicated that a sample size of 120 would give a better than 80% probability of detecting a between-group difference on the primary outcome. This calculation assumed an α of 0.05, a SD of 2 points, a dropout rate of 10%, non-compliance of 10% and correlation between baseline and final strength of 0.6.

All statistical analyses were performed using the principle of “intention to treat”. Linear regression was used for all outcomes including the primary outcome to determine mean between-group differences and 95% CI. These analyses are robust to assumptions of normality with large sample sizes.

Two additional analyses were run just on the primary outcome (because of its short ordinal and skewed nature) to check the robustness of our primary results. One was the centile routine in Stata (v15; Statacorp, TX, USA [26]). This was used to derive 95% CIs for median between-group differences. This makes no assumption about the normality of distribution and is based on the generalised Hodges–Lehmann median differences function. The second additional analysis on the primary outcome was an ordinal logistical regression (also known as a proportional odds or cumulative odds model) with initial strength and site of recruitment entered into the model as co-variates (using the ologit command of Stata) [27]. This calculates an averaged odds ratio (with 95% CI) and is reflective of the odds of attaining a higher score in the Treatment Group compared with the Control Group. The averaged odds ratio is derived from 10 different possible odds ratios for the data, which are the odds of attaining a strength score at 8 weeks of 2 points (the lowest score attained by participants at 8 weeks) versus 3 to 13 points, 2 or 3 points versus 4 to 13 points, 2 to 4 points versus 5 to 13 points, etc. Prior to using this approach, a likelihood-ratio test was used to ensure that data did not violate the proportional odds assumption (using the omdel command of STATA).

## Results

### Participants

**Fig. 1:**
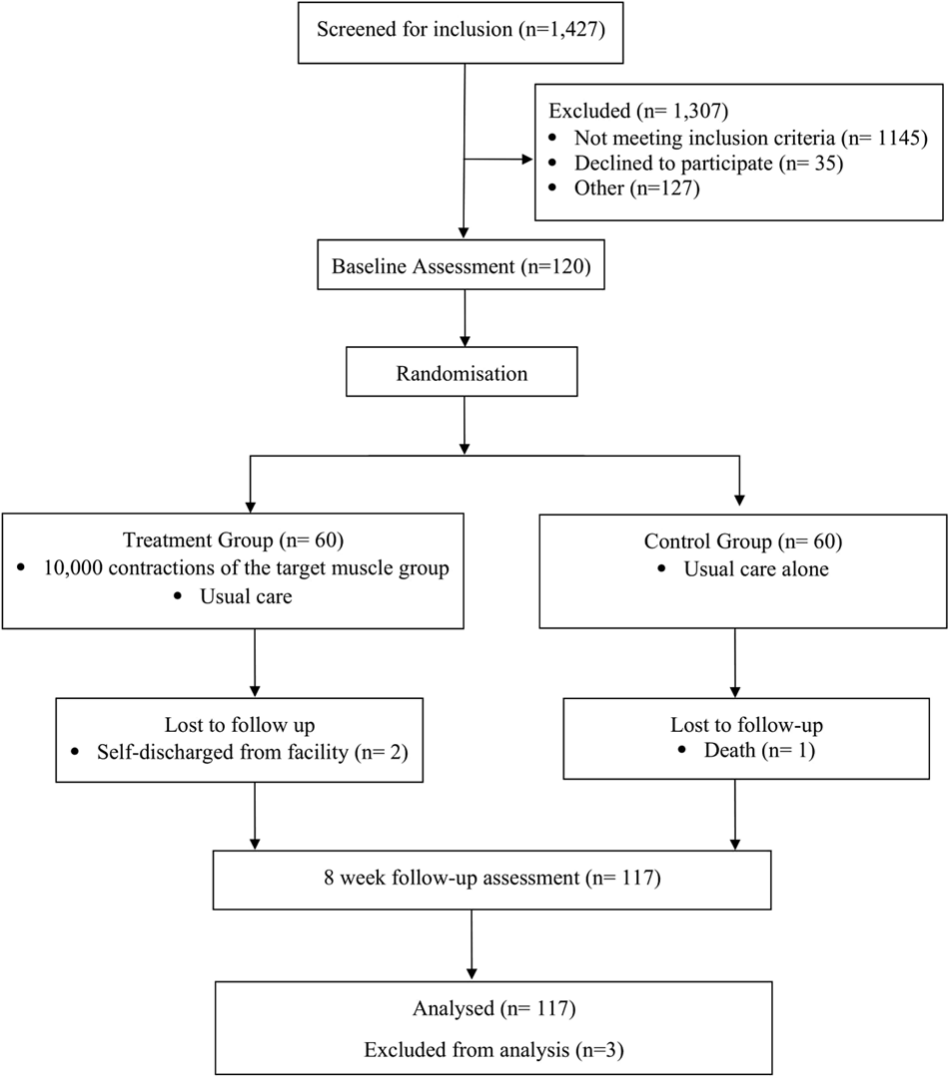
Flow of participants through the trial.

Figure 1 shows the flow of participants through the trial: 1427 participants were screened for inclusion and 120 ultimately randomised. Three participants dropped out of the trial. Two of these participants self-discharged from their facilities after less than a week of training and were unavailable for the 8-week assessments, and one participant died due to respiratory complications. The data collected for these three participants were not included in the analyses. There were no other serious adverse events although 11 participants from the Treatment Group reported minor muscle pain or fatigue after training. Some participants’ 8-week assessments were early or late with a median (interquartile range (IQR)) time between randomisation and assessment of 8.1 weeks (8.0 to 8.4).

The characteristics of participants are shown in Table 1. The median (IQR) age and time since injury were 39 years (28 to 57) and 1.9 months (1.4 to 3.1), respectively. Participants had complete and incomplete lesions with neurological levels between C1 and L4 and American Spinal Injury Association Impairment Scale (AIS) A (*n* = 36), AIS B (*n* = 19), AIS C (*n* = 40) or AIS D (*n* = 25), as defined by the International Standards for Neurological Classification of SCI. The groups were similar at baseline for most key prognostic factors.

**Table 1:** Characteristics of participants.

	Control Group (*n* = 60)	Treatment Group (*n* = 60)
Age (years), median (IQR)	39 (28 to 59)	43 (29 to 55)
Sex (F:M, *n*)	8:52	7:53
Upper extremity motor score (/50 points), median (IQR)^a^	20 (10 to 50)	18 (14 to 34)
Lower extremity motor score (/50 points), median (IQR)^a^	8 (0 to 18)	8 (0 to 22)
Type of injury		
Traumatic	57	53
Non-traumatic	3	7
Time since injury (months), median (IQR)	2.0 (1.2 to 3.4)	1.8 (1.2 to 2.8)
Neurological level^a^		
C1–4	31	38
C5–8	12	12
T1-S5	17	10
Trained muscle group, *n*		
Elbow flexors	10	5
Elbow extensors	11	16
Wrist flexors	3	5
Wrist extensors	9	16
Knee flexors	6	8
Knee extensors	11	6
Ankle dorsiflexors	10	3
Ankle plantar flexors	0	1
AIS classification, *n*^a^		
A	18	18
B	10	9
C	22	18
D	10	15
Initial strength of selected muscle group on MMT, *n*		
1/5	4	3
1+/5	13	17
2−/5	13	5
2/5	14	12
2+/5	10	14
3−/5	6	9

^a^Based on the International Standards for the Neurological Classification of SCI.

### Outcomes

The results are shown in Table 2, and the data for each participant are provided in the Supplementary file. The mean between-group difference for the primary outcome of voluntary strength at 8 weeks was 0.4/13 points (95% CI, −0.5 to 1.4) where a positive value favours the Treatment Group. The two additional analyses (i.e., the centile and ologit routines in STATA) confirmed the robustness of our results for the primary outcome with the median (95% CI) between-group difference almost identical to the mean (95% CI) between-group difference, and with neither analysis demonstrating a significant between-group difference (the details of these analyses are not reported).

**Table 2:** The mean (SD) pre and post-data, and the mean (95% CI) between-group differences for the four outcome measures.

	Control Group (*n* = 59)		Treatment Group (*n* = 58)		Between-group differences (*n* = 117)
Outcome	Baseline	Week 8	Baseline	Week 8	
Maximal voluntary strength, 0–13 points	3.5 (1.4)	6.5 (3.0)	3.7 (1.6)	7.0 (2.7)	0.4 (−0.5 to 1.4)
Participants’ perceptions of change in strength, −7 to +7 points	–	9.5 (1.6)	–	10.3 (1.7)	0.8 (0.1 to 1.4)
Participants’ perceptions of change in function, −7 to +7 points	–	9.2 (1.8)	–	9.7 (1.8)	0.6 (0.0 to 1.3)
Difference between predicted final strength and measured final strength, 0–13 points	–	−1.0 (2.6)	–	−0.7 (2.6)	0.3 (−0.6 to 1.3)

None of the between-group differences for the secondary outcomes indicated a treatment effect. The mean between-group difference for participants’ perception of change in strength and function were 0.8/15 points (95% CI, 0.1 to 1.4) and 0.6/15 points (95% CI, 0.0 to 1.3), respectively, and for therapists’ prediction of final strength was 0.3/13 points (95% CI −0.6 to 1.3), where positive values favour the Treatment Group.

The median (IQR) rating for the burden of the training undertaken by the Treatment Group was 3/10 points (0 to 5), where a score of 10 indicates “*extremely burdensome*”. All assessors remained blinded except on one occasion.

### Adherence to the training protocol

The protocol dictated that participants in the Treatment Group were supervised while performing 200 contractions per day, at least 6 days a week for 8 weeks (that is 9600 contractions over 48 training sessions). A small number of supervised training sessions were missed for various reasons including public holidays, illness, surgery and unexpected discharge from hospital. Additional sessions were provided to make up for missed sessions where possible. Two participants in the Treatment Group were discharged prior to the 8-week assessment and could not return for outpatient therapy. Instead, family members were trained and provided the therapy. In practice, participants received a median (IQR) of 48 (44 to 51) supervised training sessions in which they performed a median (IQR) of 9600 (8800 to 10,040) contractions. Participants reported doing a median (IQR) of 4 (1 to 18) additional unsupervised sessions over 8 weeks in which they performed a median (IQR) of 450 (0–2400) contractions. So, in total, participants performed a median (IQR) of 53 (47 to 65) supervised and unsupervised sessions, with a median (IQR) of 10,690 (9400 to 12,000) contractions.

## Discussion

This study indicates that 10,000 isolated contractions of very weak muscles in people with SCI over 8 weeks has either no or a very small effect on voluntary strength. The 95% CI associated with the mean between-group difference for voluntary strength spanned from −0.5 to 1.4 point on the 13-point manual muscle test indicating that the point estimate is very precise, and hence the sample size was more than adequate. However, the clinical interpretation of this result is more nuanced because while the mean between-group difference is less than our minimally worthwhile treatment effect of one point, the upper end of the 95% CI is not. It spans up to 1.4 points. Most would probably conclude that these results indicate no effect. The exception would be those who believe that a treatment effect of 1.4 points (on a 13-point manual muscle test) is clinically important. In this case, our results neither rule in nor rule out the possibility of a treatment effect. The minimally worthwhile treatment effect is somewhat arbitrary and depends on people’s perspectives. Our decision to set the minimally worthwhile treatment effect at one point was based on clinical consensus after taking into account the burden of the treatment for both participants and staff, and the implications of gains in strength for people with SCI. Others may disagree in which case their interpretation of the results would need to be adjusted accordingly. Importantly, the decision about the size of our minimally worthwhile treatment effect was made prior to commencement of the study to ensure that we were not influenced by the results per se.

Our results cannot be dismissed by criticisms of our primary outcome measure: the 13-point manual muscle test. We used this rather than more sophisticated dynamometers because we needed an outcome measure that was easy to administer and was readily interpretable. We acknowledge that the 13-point manual muscle test is less objective than dynamometry but it has clear validity and contrary to what is often assumed, it is surprisingly reliable when used by trained therapists [24]. The precision of our estimate confirms this. In addition, the assessors were blinded, which minimises bias and ensures any errors and noise in the outcome measure are randomly distributed across both groups. Therefore, if the treatment was effective, it should have been possible to demonstrate this on the 13-point manual muscle test.

The results of our secondary outcomes support the results of our primary outcome. The participants’ perceptions of improvement in function, and the therapists’ predictions of final strength did not differ between the two groups. Interestingly, the only exception was the participants’ perception of change in strength. Participants in the Treatment Group perceived that their strength improved more than those in the Control Group. However, participants were not blinded so their perceptions may in part reflect preconceived ideas about the benefits of the 10,000 additional contractions.

We included therapists’ predictions of final strength as an outcome because we wanted therapists to estimate how much stronger they thought participants would get over the 8-week period. These predictions were made before randomisation so therapists did not know whether participants would or would not receive the extra training. We then derived a value reflecting the difference between what therapists predicted and what participants achieved. These values were compared between groups. We found no between-group difference. This does not indicate that participants necessarily achieved what therapists predicted (and nor should the results be used to examine this issue). However, it does indicate that there was no systematic difference between the two groups. This type of measure is not commonly used but we believe it should be considered by future trialists and could be used with many different outcomes, not just strength. It is however important that all predictions are made prior to randomisation. The advantage of this type of outcome measure is that it provides a way for therapists to use their clinical judgement to take into account all possible factors that may influence a participant’s progress. In this way, it is somewhat similar to the Goal Attainment Scale where a five-point scale is used to determine outcomes based on predictions of future function [28].

There are many possible explanations as to why we did not find a convincing treatment effect. First, there is the possibility that greater gains in muscle strength can be achieved when training is performed within the context of a functional skill such as gait training or upper limb training although our review of the evidence does not support this hypothesis [5]. Second, our result could be explained by an insufficient training period or dosage. Ten thousand contractions over 8 weeks may not be sufficient to increase strength in the very weak muscles of people with SCI. We chose 10,000 contractions because that was the most that we could reasonably expect of participants (and therapists) on top of their usual care. Achieving more contractions than this would prove very difficult and even if it could be achieved within a trial it may not be achievable within the constraints of clinical practice particularly if many muscles needed to be trained.

The 10,000 contractions participants performed in our trial are not dissimilar to the number of contractions typically achieved when patients walk on treadmills or the like. For example, 20 min of walking, three times a week for 8 weeks at a speed of 0.3 m/s equates to 11,904 steps (or contractions). This is also in line with the number of steps provided in animal locomotor studies which is typically anywhere from a couple of hundred to a couple of thousand repetitions [20]. Our results suggest from a mechanistic perspective that any improvements in gait from these types of interventions may not merely be due to increases in strength from repeated contractions. The results of this study highlight the need for a better understanding of the responsiveness of very weak muscles to any type of training programme that involves high numbers of muscle contractions.

Importantly, both groups got stronger (Table 2). This points to one of the major problems of conducting research in this area. That is the problem of determining the effect of treatments over and above natural recovery [29], and over and above any effect of usual care. Both groups in our trial received usual care. This was the type of care typically provided by the SCI units in the study, including training for activities of daily living (e.g., training to transfer, walk, roll and push a wheelchair), as well as other forms of therapy appropriate for managing fitness and other impairments. Usual care would have invariably involved some voluntary contractions of the target muscle group, and therefore it cannot be concluded from this study that repeated contractions of any muscle groups have no benefit whatsoever. This study can only answer the question of whether a very intensive programme of 10,000 repetitions has an added benefit over and above usual care when compared with usual care alone.

In all, the results of this study raise many questions about the responsiveness of very weak muscles to training and about current physiotherapy practice. The type of training provided in this study is commonly administered in SCI units around the world, although the dosage may often be less than provided in this study. Yet the 95% CI around our point estimate for voluntary strength indicates no or very little effect. Given the importance of strength to the future independence of people with SCI, more research needs to be directed at identifying effective ways of increasing strength in the very weak muscles of these people.

## Supplementary information

Trial data for sharing

Trial data for sharing codebook

## Data Availability

All participant level data are available in the supplementary file.
