# Supplementary material for: The effects of 10,000 voluntary contractions over 8 weeks on the strength of very weak muscles in people with spinal cord injury: a randomised controlled trial
Source: Spinal Cord. 2020 Feb 21;58(8):857–64. doi: 10.1038/s41393-020-0439-1 (PMC7402990; doi:10.1038/s41393-020-0439-1)
Supplement: Supplementary file 1 — Trial data for sharing [file 41393_2020_439_MOESM1_ESM.pdf]

rand_grp_a	demo_site	demo_gen	demo_time	demo_age	demo_asia	demo_asia	demo_asia	demo_asia	baseline_ta	baseline_m	baseline_tl	eightwk_m	eightwk_pp
control	CRP	Male	2.365552	40.82466	50	14	L1	C	Knee exter	2 Able to m	4 Able to m	2 Able to m	8
experiment	CRP	Male	2.95694	27.01644	0	14	C4	C	Ankle dorsi	2+ Able to	4+ Able to	4+ Able to	11
experiment	CRP	Male	4.56683	60.85753	27	18	C2	D	Wrist flexor	1+ Able to	3+ Able to	2+ Able to	8
experiment	CRP	Male	1.379905	38.85205	18	0	C4	A	Wrist exter	2+ Able to	4+ Able to	4+ Able to	11
experiment	CRP	Male	2.102713	35.85205	36	34	C4	D	Elbow exte	2 Able to m	4 Able to m	2 Able to m	9
control	CRP	Male	1.54418	22.87945	50	7	T11	C	Ankle dorsi	1+ Able to	3+ Able to	4+ Able to	12
control	CRP	Male	1.708454	50.90137	27	17	C3	D	Elbow exte	2 Able to m	4 Able to m	4 Able to m	8
control	CRP	Male	4.632539	35.89041	50	25	T3	A	Knee flexor	2 Able to m	4 Able to m	4+ Able to	9
control	CRP	Female	0.755662	20.10685	44	18	C6	D	Knee exter	2 Able to m	4+ Able to	3+ Able to	9
experiment	CRP	Male	2.365552	20.59178	14	0	C4	A	Elbow exte	2 Able to m	4 Able to m	2 Able to m	8
experiment	CRP	Male	3.482618	21.24657	4	0	C4	A	Elbow flexo	2 Able to m	4- Able to r	4- Able to r	8
control	CRP	Male	1.281341	61.01096	19	34	C2	D	Wrist exter	2- Able to r	3+ Able to	2+ Able to	8
control	CRP	Male	2.562681	28.15891	50	5	T12	C	Knee exter	3- Able to r	4+ Able to	4- Able to r	10
experiment	CRP	Male	2.299842	55.18904	7	0	C2	A	Elbow exte	1+ Able to	3+ Able to	2 Able to m	8
experiment	CRP	Male	0.821372	55.17534	23	0	C6	A	Elbow exte	2 Able to m	3+ Able to	2 Able to m	6
control	CRP	Male	1.182776	27.20274	50	5	T10	C	Knee exter	2+ Able to	4 Able to m	4+ Able to	11
experiment	CRP	Male	0.887082	17.65206	16	0	C2	A	Elbow exte	2 Able to m	4+ Able to	2 Able to m	9
control	CRP	Male	2.135568	30.29589	15	0	C4	A	Elbow exte	2 Able to m	4+ Able to	3- Able to r	10
control	CRP	Male	1.281341	45.30411	27	18	C4	C	Ankle dorsi	2+ Able to	4- Able to r	4+ Able to	10
experiment	CRP	Male	1.774164	55.31507	30	41	C4	D	Wrist exter	1+ Able to	3+ Able to	3+ Able to	9
experiment	CRP	Male	1.839874	18.29041	6	0	C4	A	Wrist exter	2+ Able to	3+ Able to	4+ Able to	10
experiment	CRP	Male	2.102713	45.40274	16	0	C4	A	Wrist exter	1+ Able to	3+ Able to	3+ Able to	9
control	CRP	Male	2.168423	28.40548	50	8	T12	A	Ankle dorsi	1+ Able to	4- Able to r	4- Able to r	10
experiment	CRP	Male	1.54418	48.41644	11	6	C5	C	Wrist exter	1+ Able to	3+ Able to	4- Able to r	12
experiment	CRP	Male	0.689953	58.37534	21	35	C5	D	Knee exter	2+ Able to	4+ Able to	5 Normal s	13
control	CRP	Male	1.182776	35.34521	10	0	C3	A	Wrist exter	2- Able to r	3+ Able to		
experiment	CRP	Male	2.661246	24.54795	27	0	C5	A	Wrist flexor	2+ Able to	4 Able to m	4- Able to r	10
control	CRP	Male	3.384054	18.54795	3	0	C4	A	Elbow flexo	2 Able to m	3 Able to m	2+ Able to	8
control	CRP	Male	1.905584	33.55616	9	16	C4	C	Knee exter	2+ Able to	4+ Able to	4- Able to r	12
control	CRP	Male	1.445615	28.54521	50	11	T12	C	Ankle dorsi	1+ Able to	3+ Able to	3+ Able to	11
experiment	CRP	Male	1.34705	26.55616	50	12	T1	C	Knee exter	2+ Able to	4+ Able to	4+ Able to	12
control	CRP	Male	1.60989	30.5589	22	10	C3	C	Knee exter	2+ Able to	4+ Able to	4 Able to m	10
experiment	CRP	Male	1.839874	17.69589	14	0	C4	A	Wrist exter	1+ Able to	3+ Able to	2- Able to r	8
experiment	CRP	Male	1.445615	30.18904	23	26	C4	D	Wrist exter	1 Visible o	3+ Able to	4+ Able to	11
control	CRP	Male	0.755662	38.71233	19	0	C4	B	Elbow exte	2 Able to m	4 Able to m	2+ Able to	11

control	CRP	Male	2.398407	25.73699	50	29	T4	D	Knee exter	3- Able to r	4+ Able to	4 Able to m	11
control	CRP	Male	0.657098	35.7863	2	16	C4	B	Elbow flexo	2 Able to m	4- Able to r	4- Able to r	12
control	CRP	Male	0.394259	35.80548	5	12	C4	B	Elbow flexo	2 Able to m	4- Able to r	4- Able to r	10
control	CRP	Male	4.074006	27.8137	9	0	C4	A	Wrist exter	1 Visible o	3 Able to m	2- Able to r	8
experiment	CRP	Male	0.985647	50.87671	9	11	C3	C	Knee exter	2- Able to r	4+ Able to	4+ Able to	13
experiment	CRP	Male	1.971293	30.86575	50	8	T10	A	Knee exter	1 Visible o	3+ Able to	4+ Able to	12
experiment	CRP	Male	1.051356	50.95617	17	49	C4	D	Elbow exte	2 Able to m	4- Able to r	4- Able to r	10
control	CRP	Male	1.117066	55.9726	11	3	C3	C	Elbow exte	2 Able to m	4 Able to m	3 Able to m	9
experiment	GDW	Female	4.402555	67.34247	33	40	C4	D	Knee flexor	3- Able to r	3 Able to m	3- Able to r	10
control	GDW	Male	3.384054	45.36164	50	8	T12	A	Knee exter	3- Able to r	3 Able to m	3- Able to r	10
experiment	GDW	Male	5.158217	47.62466	50	16	T10	C	Knee flexor	2+ Able to	3+ Able to	2+ Able to	11
control	GDW	Male	2.924085	31.56164	50	16	T12	C	Knee flexor	2- Able to r	3+ Able to	3- Able to r	9
control	GDW	Male	2.299842	57.88219	2	0	C2	A	Elbow flexo	2 Able to m	3 Able to m	4- Able to r	10
experiment	GDW	Male	3.942587	32.15342	13	0	C4	B	Elbow exte	2 Able to m	3 Able to m	2+ Able to	9
experiment	GDW	Male	2.332697	54.90685	13	22	C4	C	Elbow exte	3- Able to r	3+ Able to	3- Able to r	12
experiment	GDW	Male	4.895379	61.00274	9	18	C4	C	Elbow exte	2- Able to r	3- Able to r	3- Able to r	12
control	GDW	Female	4.829669	25.02466	50	25	L3	C	Ankle dorsi	1+ Able to	2 Able to m	2- Able to r	8
experiment	GDW	Male	0.788517	53.79726	18	0	C5	A	Elbow exte	2 Able to m	3- Able to r	3 Able to m	10
control	ISIC	Male	4.599685	46.48219	9	0	C4	A	Wrist exter	2+ Able to	4 Able to m	2+ Able to	8
experiment	ISIC	Male	2.037003	20.71233	14	0	C4	C	Wrist exter	3- Able to r	4 Able to m	3+ Able to	9
control	ISIC	Male	0.788517	39.03836	16	0	C5	B	Elbow exte	2- Able to r	3 Able to m	2+ Able to	9
experiment	ISIC	Male	1.149921	45.34246	16	0	C5	A	Wrist exter	3- Able to r	4- Able to r		
control	ISIC	Male	1.938439	59.96164	3	0	C4	A	Elbow flexo	2+ Able to	4 Able to m	3- Able to r	8
experiment	ISIC	Male	1.445615	35.15068	17	0	C5	B	Wrist exter	2+ Able to	4 Able to m	4 Able to m	11
control	ISIC	Male	4.43541	37.30685	10	0	C4	B	Elbow flexo	2 Able to m	4 Able to m	2+ Able to	8
experiment	ISIC	Male	4.533975	33.11507	50	9	L1	C	Ankle plant	3- Able to r	5 Normal s	3+ Able to	12
experiment	ISIC	Male	4.599685	47.11233	4	0	C4	A	Elbow flexo	2- Able to r	3+ Able to	3 Able to m	12
control	ISIC	Male	4.632539	19.95617	20	0	C6	A	Wrist flexor	2 Able to m	3+ Able to	3- Able to r	9
experiment	ISIC	Male	2.431262	36.27671	22	40	C5	D	Wrist exter	2+ Able to	3+ Able to	3 Able to m	11
control	ISIC	Male	3.942587	24.1726	20	0	C6	B	Elbow exte	1+ Able to	3- Able to r	2 Able to m	10
control	ISIC	Male	2.464117	36.49041	13	0	C5	A	Elbow exte	2+ Able to	3+ Able to	3 Able to m	11
experiment	ISIC	Male	2.266987	34.95068	4	0	C4	A	Elbow flexo	1+ Able to	1+ Able to	2 Able to m	10
control	ISIC	Male	1.971293	28.35342	8	0	C5	A	Wrist exter	3- Able to r	4 Able to m	3 Able to m	8
experiment	ISIC	Male	5.683896	26.89863	50	14	L1	B	Knee exter	2 Able to m	3- Able to r		
control	ISIC	Male	2.365552	21.43014	16	0	C5	A	Elbow exte	1+ Able to	2 Able to m	1+ Able to	8
experiment	PAH	Male	-3.41691	41.99452	26	33	C2	D	Ankle dorsi	1+ Able to	2- Able to r	1+ Able to	12

control	PAH	Female	4.43541	46.07123	27	1	C6	C	Ankle dorsi	1+ Able to	2 Able to m	1+ Able to	7
control	PAH	Male	4.205426	58.39452	29	6	C6	C	Knee exter	1 Visible o	2- Able to r	3- Able to r	13
experiment	PAH	Male	3.08836	72.89041	4	8	C4	C	Wrist exter	1 Visible o	1+ Able to	3- Able to r	7
experiment	PAH	Female	1.741309	64.09041	50	13	T10	C	Knee exter	2+ Able to	3+ Able to	1+ Able to	8
control	PAH	Male	2.365552	54.53425	20	30	C4	C	Ankle dorsi	3- Able to r	4 Able to m	1+ Able to	12
experiment	POWH	Male	2.431262	64.47123	14	0	C4	A	Wrist exter	2- Able to r	3- Able to r	2+ Able to	10
control	POWH	Male	4.238281	21.32877	10	0	C4	A	Elbow flexo	2- Able to r	2+ Able to	3 Able to m	11
control	POWH	Female	0.919937	62.51233	50	18	L2	C	Knee flexor	2 Able to m	3 Able to m	2 Able to m	9
experiment	POWH	Female	1.117066	79.79726	50	8	T2	C	Knee flexor	3- Able to r	4+ Able to	3+ Able to	12
experiment	POWH	Male	1.182776	31.65479	28	38	C6	D	Knee flexor	1+ Able to	4 Able to m	4- Able to r	11
control	POWH	Male	1.741309	39.09041	20	0	C6	A	Wrist flexor	2- Able to r	3- Able to r	2+ Able to	8
experiment	POWH	Female	1.60989	62.39178	50	43	L1	D	Knee flexor	1+ Able to	3+ Able to	4- Able to r	12
experiment	POWH	Male	3.482618	35.68219	50	8	T12	C	Knee flexor	1+ Able to	2+ Able to	2- Able to r	8
control	POWH	Male	3.646893	20.86849	45	28	C1	D	Knee flexor	2+ Able to	3+ Able to	4- Able to r	10
control	POWH	Female	3.154069	72.27671	43	34	C5	D	Ankle dorsi	1+ Able to	2 Able to m	1+ Able to	6
experiment	POWH	Male	4.172571	27.30411	50	5	T6	C	Ankle dorsi	1+ Able to	2+ Able to	4- Able to r	11
experiment	POWH	Male	1.117066	55.1863	11	21	C4	C	Elbow exte	2 Able to m	3+ Able to	3+ Able to	12
experiment	POWH	Male	1.675599	22.50685	6	0	C4	B	Elbow flexo	2+ Able to	3 Able to m	3 Able to m	11
control	POWH	Male	1.314196	58.98082	50	26	T11	D	Knee flexor	1 Visible o	2 Able to m	1+ Able to	9
control	POWH	Male	3.581183	69.34521	7	4	C4	C	Ankle dorsi	1+ Able to	1+ Able to	2- Able to r	7
control	POWH	Male	3.318344	48.97808	12	17	C4	C	Elbow flexo	1+ Able to	2+ Able to	1+ Able to	7
experiment	POWH	Male	1.807019	73.67397	43	34	C4	D	Knee flexor	2+ Able to	3 Able to m	3- Able to r	9
control	RNSH	Female	0.821372	22.95342	50	2	T3	B	Ankle dorsi	2- Able to r	4 Able to m	4+ Able to	13
control	RNSH	Male	1.149921	59.33151	14	18	C4	C	Elbow exte	2- Able to r	2+ Able to	4 Able to m	10
experiment	RNSH	Male	0.624243	50.33151	21	0	C4	C	Wrist flexor	2+ Able to	3+ Able to	3- Able to r	12
experiment	RNSH	Male	0.788517	29.48219	23	0	C4	B	Wrist flexor	2- Able to r	2+ Able to	2+ Able to	8
experiment	RNSH	Female	0.919937	28.49863	23	0	C6	B	Wrist flexor	1+ Able to	2 Able to m	2- Able to r	11
control	RNSH	Male	1.577035	59.76986	5	4	C4	C	Elbow exte	1+ Able to	2- Able to r	2- Able to r	8
experiment	RNSH	Male	1.41276	28.39452	14	0	C4	B	Elbow exte	1+ Able to	2- Able to r	2 Able to m	9
control	RNSH	Female	0.854227	21.20274	50	4	T11	A	Knee exter	2+ Able to	3- Able to r	4 Able to m	11
control	RNSH	Male	0.788517	22.15891	50	17	L1	C	Knee exter	2- Able to r	3 Able to m	4+ Able to	12
control	RNSH	Male	1.511325	67.00822	39	29	C4	D	Knee flexor	1+ Able to	2 Able to m	1+ Able to	13
experiment	RNSH	Male	1.511325	43.30959	14	0	C4	B	Wrist exter	3- Able to r	3 Able to m	4 Able to m	12
experiment	RNSH	Male	0.854227	25.41096	14	0	C4	B	Wrist exter	3- Able to r	3 Able to m	4- Able to r	12
control	RNSH	Male	0.821372	67.91233	34	11	C4	C	Elbow exte	2- Able to r	2+ Able to	2 Able to m	8
control	RNSH	Male	1.182776	72.82466	16	0	C3	B	Wrist exter	2- Able to r	2+ Able to	3- Able to r	9

control	RNSH	Female	0.558533	66.40548	30	32	C2	D	Wrist exter	3- Able to r	3+ Able to	4- Able to r	9
experiment	RNSH	Male	0.591388	50.66575	18	18	C4	C	Elbow exte	1+ Able to	2 Able to m	4+ Able to	12
control	RNSH	Male	0.854227	45.14795	24	0	C6	A	Wrist flexor	2- Able to r	3- Able to r	3+ Able to	9
control	RNSH	Male	0.591388	74.98356	21	28		D	Wrist exter	2- Able to r	3+ Able to	4- Able to r	10
control	RR	Male	5.125363	60.00274	14	34	C4	C	Elbow flexo	2+ Able to	3+ Able to	4- Able to r	10
experiment	RR	Female	1.675599	73.3726	35	41	C5	D	Elbow exte	3- Able to r	3+ Able to	4- Able to r	12
experiment	RR	Male	2.069858	71.77261	14	23	C4	C	Elbow exte	1+ Able to	2 Able to m	2- Able to r	10
experiment	RR	Male	2.858375	52.7863	36	19	C2	D	Knee flexor	1+ Able to	2+ Able to	2+ Able to	11
experiment	RR	Male	2.726956	32.70137	3	0	C4	A	Elbow flexo	2 Able to m	3 Able to m	2+ Able to	10
control	RR	Male	2.661246	75.96165	5	11	C4	B	Elbow flexo	1 Visible o	2- Able to r	1+ Able to	8
experiment	RR	Female	2.989795	29.92877	41	28	C4	D	Elbow exte	2 Able to m	3 Able to m	3- Able to r	10
experiment	RR	Male	2.759811	54.9589	16	0	C5	A	Wrist exter	2+ Able to	3 Able to m	3- Able to r	7
control	RR	Male	3.909732	57.85479	4	0	C4	B	Wrist exter	1+ Able to	2+ Able to	2- Able to r	10

eightwk_pr	eightwk_bh_coded
10	10
7	11
7	8
10	10
9	9
11	11
10	9
10	9
13	13
8	8
8	9
9	8
10	10
8	9
6	7
11	11
9	9
7	8
9	10
8	11
7	11
8	10
11	12
10	12
13	13
10	10
7	9
9	12
11	11
11	12
11	11
8	8
11	11
10	12

10	11
11	11
9	10
8	8
13	13
10	11
10	10
9	9
10	10
8	10
10	11
10	10
8	10
9	12
10	12
9	9
7	7
11	11
7	8
8	9
9	9
8	8
10	11
8	8
12	12
11	11
7	12
12	12
10	9
11	11
7	9
8	8
9	8
10	12

7	7
7	12
7	8
8	10
11	11
7	11
12	12
11	9
12	
12	11
9	7
11	11
11	11
11	11
6	6
11	11
12	11
11	10
9	9
9	7
7	7
12	7
12	13
11	6
12	11
8	8
10	10
7	7
9	9
8	10
12	13
13	12
10	11
10	11
7	7
9	7

9	10
12	12
10	10
7	10
10	10
12	12
10	11
12	10
9	9
7	7
10	7
7	7
7	10
