# Supplementary material for: The effects of 10,000 voluntary contractions over 8 weeks on the strength of very weak muscles in people with spinal cord injury: a randomised controlled trial
Source: Spinal Cord. 2020 Feb 21;58(8):857–64. doi: 10.1038/s41393-020-0439-1 (PMC7402990; doi:10.1038/s41393-020-0439-1)
Supplement: Supplementary file 2 — Trial data for sharing codebook [file 41393_2020_439_MOESM2_ESM.pdf]

Variable Name	Variable Label	Answer Label	Answer Code
rand_grp_a	group		
		control	0
		experimental	1
demo_site	Recruitment site	Open ended	
demo_gender	Sex		
		Male	1
		Female	2
demo_time_since_inj	time since injury (months)	Open ended	
demo_age	age	Open ended	
demo_asia_uems_total	UEMS total	Open ended	
demo_asia_lems_total	LEMS total	Open ended	
demo_asia_nli	Neurological level of injury		
		C1	0
		C2	1
		C3	2
		C4	3
		C5	4
		C6	5
		C7	6
		C8	7
		T1	8
		T2	9
		T3	10
		T4	11
		T5	12
		T6	13
		T7	14
		T8	15
		T9	16
		T10	17
		T11	18
		T12	19
		L1	20
		L2	21
		L3	22
		L4	23

		L5	24
		S1	25
		S2	26
		S3	27
		S4	28
		S5	29
demo_asia_ais	Asia Impairment Scale (AIS)		
		A	0
		B	1
		C	2
		D	3
		E	4
baseline_targetmm	Target muscle		
		Elbow flexors	1
		Elbow extensors	2
		Wrist flexors	3
		Wrist extensors	4
		Knee flexors	5
		Knee extensors	6
		Ankle dorsiflexors	7
		Ankle plantarflexors	8
baseline_mmstrength	strength at baseline		
		0 No palpable contraction	0
		or palpable contraction but unabl	1
		rough a small range (< 50%) with	2
		ugh a large range (= or >50%) wi	3
		rough full range of (=100%) with	4
		e through a small range (< 50%)	5
		through a large range (= or >50%	6
		ve through full range (=100%) ag	7
		00%) against gravity with small re	8
		100%) against gravity with small r	9
		0%) against gravity with moderate	10
		e (=100%) against gravity with ma	11
		5 Normal strength	12
baseline_therapistpredict	predicted strength at baseline		
		0 No palpable contraction	0

		or palpable contraction but unabl	1
		rough a small range (< 50%) with	2
		ugh a large range (= or >50%) wi	3
		rough full range of (=100%) with	4
		e through a small range (< 50%)	5
		through a large range (= or >50%	6
		ve through full range (=100%) ag	7
		00%) against gravity with small re	8
		100%) against gravity with small r	9
		0%) against gravity with moderate	10
		ge (=100%) against gravity with ma	11
		5 Normal strength	12
eightwk_mmstrength	strength at 8 weeks		
		0 No palpable contraction	0
		or palpable contraction but unabl	1
		rough a small range (< 50%) with	2
		ugh a large range (= or >50%) wi	3
		rough full range of (=100%) with	4
		e through a small range (< 50%)	5
		through a large range (= or >50%	6
		ve through full range (=100%) ag	7
		00%) against gravity with small re	8
		100%) against gravity with small r	9
		0%) against gravity with moderate	10
		e (=100%) against gravity with ma	11
		5 Normal strength	12
eightwk_ppstrength_coded	rceptions of strength on 15 points sca	Open ended	
eightwk_ppf_coded	rceptions of function on 15 points sca	Open ended	
eightwk_bh_coded	eption of benefits/harns on 15 points s	Open ended	

**Variable Type**
Numeric
String
Numeric
Numeric
Numeric
Numeric
Numeric
Numeric

Numeric

Numeric

Numeric

Numeric

Numeric

Numeric
Numeric
Numeric
